# The Role of Immunotherapy in Pancreatic Cancer

**DOI:** 10.3390/curroncol29100541

**Published:** 2022-09-23

**Authors:** Reetu Mukherji, Dipanjan Debnath, Marion L. Hartley, Marcus S. Noel

**Affiliations:** 1 The Ruesch Center for the Cure of Gastrointestinal Cancers, Georgetown Lombardi Comprehensive Cancer Center, Division of Hematology and Oncology, Medstar Georgetown University Hospital, 3800 Reservoir Road NW, Washington, DC 20007, USA; 2 Department of Internal Medicine, Medstar Washington Hospital Center, 110 Irving Street NW, Washington, DC 20010, USA

**Keywords:** immunotherapy, PD-1, PD-L1, pancreas cancer, adenocarcinoma, checkpoint inhibitor, vaccine

## Abstract

Pancreatic adenocarcinoma remains one of the most lethal cancers globally, with a significant need for improved therapeutic options. While the recent breakthroughs of immunotherapy through checkpoint inhibitors have dramatically changed treatment paradigms in other malignancies based on considerable survival benefits, this is not so for pancreatic cancer. Chemotherapies with modest benefits are still the cornerstone of advanced pancreatic cancer treatment. Pancreatic cancers are inherently immune-cold tumors and have been largely refractory to immunotherapies in clinical trials. Understanding and overcoming the current failures of immunotherapy through elucidating resistance mechanisms and developing novel therapeutic approaches are essential to harnessing the potential durable benefits of immune-modulating therapy in pancreatic cancer patients.

## 1. Introduction

Continued dismal survival outcomes in patients with pancreatic adenocarcinoma (PDAC) are driving major efforts today to identify novel treatments for this high-risk group. In the United States, although PDAC is estimated to be the tenth most diagnosed cancer, with 62,210 new cases in 2022, it is highly lethal and estimated to be the third leading cause of cancer death [1]. Despite our advances in curative-intent surgery, radiation, and chemotherapy, the five-year overall survival (OS) rates are only 43.9% for patients diagnosed with early-stage, localized PDAC. In addition, most patients are diagnosed with regional or incurable distant metastases, both of which are associated with significantly worse five-year OS rates of 14.7% and 3.1%, respectively. The current standard front-line treatments for advanced disease in patients with decent performance status are combination chemotherapy with either the modified FOLFIRINOX regimen (5-FU, leucovorin, oxaliplatin, and irinotecan) or combination gemcitabine plus nab-paclitaxel, based on results from the phase 3 PRODIGE 4 and MPACT trials, respectively [2,3]. There are limited subsequent therapy options (only one with Food and Drug Administration [FDA] approval), most of which are chemotherapies with modest benefit. As immune-oncology therapies (IO)—those that modulate a patient’s ability to generate an anti-tumor immune response—have revolutionized the cancer field across multiple solid tumors, their evaluation in PDAC has not been fruitful.

Several forms of immunotherapy have been developed over the years, such as immunostimulatory cytokines, oncolytic viruses, adoptive cell transfer, and tumor-targeting (bi-specific) antibodies, all of which work by enhancing the existing immune system’s anti-neoplastic effect [4]. Of all the immunotherapies, monoclonal antibodies (mAbs) that inhibit immunosuppressive signals on cancer or immune cells, also known as immune checkpoint inhibitors (ICI), are the most used in clinical practice today, with multiple US FDA approvals across solid tumors. By modulating the patient’s immune system’s response, these therapies have demonstrated exciting, durable benefits in subsets of patients. In gastrointestinal (GI) cancers specifically, ICI have led to survival benefits in the adjuvant setting in upper GI tumors (UGI); advanced setting when combined with chemotherapy or when used alone in UGI tumors, especially in tumors with higher programmed death-ligand 1 expression; advanced setting when combined with chemotherapy in biliary tract tumors; and advanced setting as monotherapy or combined with anti-angiogenesis therapy or a second ICI in hepatocellular carcinoma [5,6,7,8,9,10,11,12,13,14,15]. In colorectal cancers, they benefit a subgroup of patients whose tumors harbor predictive biomarkers of microsatellite-high (MSI-H) or mismatch repair deficiency (dMMR) status [16,17,18]. In addition, ICI have tumor-agnostic approvals for patients with predictive tumor biomarkers of MSI-H/dMMR status or tumor mutation burden (TMB) ≥ 10 [19,20]. Unfortunately, as with other IO approaches, we have seen disappointing results with ICI in patients with PDAC to date.

However, researchers are beginning to uncover signals pointing to subsets of PDAC patients that may benefit from IO based on subgroup and translational exploratory analyses. Identifying improved predictive biomarkers is an area of high research interest. Additionally, it is hoped that mechanisms of resistance will be defined in ongoing tumor microenvironment (TME) investigations. Myriad studies of novel therapeutics attempting to overcome these barriers or increase tumor immunogenicity are underway. While we have not yet harnessed the full potential of IO in PDAC, we remain hopeful for the future. Herein, we will review the current understanding and potential future directions of IO in PDAC.

## 2. Immune Checkpoint Inhibitors

### 2.1. Introduction to ICI

Cancers may be distinguished from normal cells by the adaptive immune system, which occurs through frequent genetic alterations resulting in diverse antigen expression. T-cell receptors (TCR) recognize these unique cancer antigens bound to major histocompatibility complexes (MHC) on antigen-presenting cells (APCs). These events signal a cascade of T-cell activation, clonal proliferation of antigen-specific cells, recruitment of immune effector cells, cytokine release, and eventual cytotoxic T-cell-mediated tumor cell death [21]. However, these steps are regulated by a balance of costimulatory and inhibitory molecular interactions (or immune checkpoints) between T cells and APCs. Normally, the presence of inhibitory signaling is crucial for self-tolerance and protecting normal bystander tissue from auto-immune targeting. However, tumor cells capitalize on this mechanism and can generate immunosuppressive environments and evade immune attack by upregulating inhibitory and downregulating costimulatory signaling.

ICI exhibit their anti-tumor effect by blocking inhibitory signaling checkpoints, so T-cell-mediated immune responses may proceed unhindered. The introduction of ICI has undoubtedly been the foremost oncologic achievement of the past decade. Related US FDA approvals started with ipilimumab for melanoma in 2011, after which 8 additional ICI approvals across multiple cancer types ensued (Table 1).

Numerous inhibitory checkpoint immunoreceptors have been studied and targeted [22]. One example is cytotoxic T-lymphocyte-associated antigen 4 (CTLA-4), which is a receptor expressed on T cells. Normally after T-cell antigen recognition, CD28, a costimulatory molecule expressed on T cells, binds to CD80 or CD86 on APCs and amplifies TCR signaling and T-cell activation [23]. This signaling is balanced by CTLA-4 by outcompeting CD28 binding given its higher affinity for CD80/86 and then reducing CD80/86 cell surface expression, ultimately limiting CD28-mediated immune activation [24]. Ipilimumab and another ICI tremelimumab are IgG mAbs that successfully target CTLA-4 [25,26].

Other well-studied inhibitory checkpoint proteins are programmed cell death protein 1 (PD-1) and PD-L1. PD-1 expressed on T cells interacts with PD-L1, typically expressed on APCs and tumor cells, leading to inhibitory signaling primarily through targeting CD28 [27]. mAbs against PD-1, PD-L1, and CTLA-4 have FDA tumor-specific approvals across numerous non-GI and GI cancers, excluding PDAC, as previously described [28]. A novel checkpoint target, LAG-3, emerged, and in melanoma, a drug acting on LAG3, relatlimab, hailed the first non-CTLA-4, PD-1, or PD-L1 targeting approval based on the RELATIVITY-047 trial [29]. Many other ICI with novel targets are under development. The toxicity profile of established ICI differs from traditional cytotoxic chemotherapy and tends to involve off-target immune toxicities that may affect any organ system [25]. These are often managed by holding cycles of ICI or reversing the immune effects with steroids and other immunosuppressants, depending on the severity of the adverse event.

Much of the excitement around the ICI drug class stems from their potential to induce enhanced, durable responses that last for years after patients come off treatment [28,30]. Predictive biomarkers like MSI-H/dMMR status, TMB, tumor-infiltrating lymphocytes (TILs), and PD-L1 expression, among others, have been identified to stratify patient likelihood of benefiting from ICI [31]. Efficacy seen in patients with a range of tumor types that have MSI-H/dMMR and high TMB (≥10 mutations per megabase [mut/Mb]) status has led to tumor-agnostic approvals for patients with tumors that fit this molecular profile [19,20]. PD-L1 positivity or higher PD-L1 scores on tumor analysis are predictive of benefit in some tumor types but not in others [32]. In addition, measuring PD-L1 is non-standardized, with numerous assays and methods of quantification utilized across studies. Intra- and inter-tumor molecular heterogeneity in these biomarkers must also be considered. Unfortunately, our biomarkers today are not perfect, and there are ongoing efforts to identify more reliable markers to predict benefit. This is especially considered in PDAC with the hope of identifying potentially ICI-responding patients (Table 2).

### 2.2. ICI Monotherapy in PDAC

A phase 2 trial by Royal et al., published in 2010, evaluated ipilimumab monotherapy in 27 patients with advanced PDAC [33]. While two patients with locally advanced disease reportedly had minor responses, the overall response rate (ORR) by RECIST was unimpressive at 0%, and there was no improvement in OS. Of note, there was one patient who initially progressed but continued ipilimumab with an eventual clinically significant delayed response. Biomarker correlates were not reported. In 2012, a phase 1 study evaluated an anti-PD-L1 therapeutic agent (BMS-936559) in advanced PDAC patients who progressed on at least one prior line of therapy and similarly reported no responses [49]. In addition, the median OS (mOS) was dismal at four months when tremelimumab (anti-CTLA-4) was used in at least the second line setting in 20 advanced PDAC patients after prior 5-fluorouracil (5-FU) or gemcitabine-based therapy [34]. In fact, all 18 evaluable patients in this trial had progression of disease (POD).

However, in the KEYNOTE-158 phase 2 study, which only included patients with advanced MSI-H/dMMR PDAC that had progressed on prior standard-of-care (SOC) therapy (22 patients in total), the ORR was 18.2% with pembrolizumab (anti-PD-1) monotherapy [35]. This included one complete response (CR), three partial responses (PR), and a promising median duration of response (mDOR) of 13.4 months (range, 8.1 months to over 16 months). Durable responses have also been reported with ICI in an MSI-H/dMMR subgroup in a small retrospective study of 10 patients where mDOR was not reached after a median follow-up of 22.6 months [50]. Emerging studies also suggest that blood-based testing of MSI-H status may be accurate and predictive of ICI responses [51]. This may be important to consider, especially if obtaining tumor tissue for profiling is not feasible.

Collectively, while ICI monotherapy has been ineffective in microsatellite stable (MSS) PDAC to date, the clinical benefit with durable responses seen in previously treated MSI-H/dMMR PDAC patients supports our current National Comprehensive Cancer Network (NCCN) guidelines, which recommend MSI/MMR testing in advanced disease and ICI therapy for advanced treatment-refractory or treatment-intolerant patients with PDAC that has MSI-H/dMMR but not microsatellite stable (MSS) or proficient mismatch repair (pMMR) status [52].

Understanding the tumor immune microenvironment in MSS PDAC to elucidate mechanisms of IO resistance is a major area of research. Numerous studies across cancer types have demonstrated that higher levels of CD8+ TILs are associated with clinical benefit from ICI [53]. In PDAC, immunosuppressive microenvironments have lower T-cell densities in tumor epithelial compartments compared to stromal compartments. Additionally, juxta-tumoral sites have more regulatory T cells (T-reg) and macrophages that suppress effector T-cell function, and these activities likely contribute to immune evasion and IO inefficacy [54,55]. PDAC itself modulates the immune milieu through cytokines like TGF-β, chemokine receptor 5, and IL-10, which are involved in inhibiting T-cell activation and promoting T-reg differentiation, activation, and homing [56,57].

PDACs also tend to have an increased presence of myeloid-derived suppressor cells (MDSC), tumor-associated macrophage (TAM) signaling, and immunosuppressive cytokine signaling, all of which facilitate tumor growth and metastases [58,59]. Studies suggest that the microenvironment may vary between the primary pancreatic tumor and metastatic sites. For example, studies have found lower CD8+ T-cell densities and higher CD4+/CD8+ ratios in pre-treatment metastatic PDAC tumor samples compared to primary tumor samples [54]. In addition, TMB frequencies vary across cancer types. PDACs tend to have lower rates of somatic mutations, which may partly explain their low immunogenicity and responses to ICI [60]. In a retrospective analysis of over 4000 PDAC tumor samples, mutant KRAS was seen in 81% of PDAC and was associated with higher M1 macrophages and cancer-associated fibroblast infiltration and lower CD4+/CD8+, natural killer (NK) cells, MSI-H status, and TMB compared to KRAS wild-type samples. This pattern was similar but more pronounced than seen in KRAS mutant/wild-type colorectal cancer, another immune-cold tumor [61]. With these potential reasons for primary ICI resistance in mind, strategies combining ICI with other drugs to enhance immunogenicity and overcome these barriers have been explored.

### 2.3. Dual ICI Therapy in PDAC

One strategy tested to improve ICI efficacy and overcome primary resistance is combining multiple ICIs in treatment. In the first phase 2 study evaluating dual ICI therapy, 65 patients with metastatic PDAC (mPDAC) were randomized to either durvalumab (anti-PD-L1) monotherapy or durvalumab plus tremelimumab combination therapy in the second line setting [37]. The ORR was 3.1% and 0% in the combination and durvalumab monotherapy arms, respectively. The median PFS (mPFS) was 1.5 months in both arms, and mOS was also similar at 3.1 months and 3.6 months in each arm, respectively. The one patient with a confirmed PR in the combination arm had metastatic disease with low PD-L1 expression but MSI-H/dMMR status. This patient achieved a PR by week 6 and then POD at week 24, but was still alive by week 61 at the time of data cut-off.

Interestingly, in another patient with PD-L1 low and MSS status randomized to the combination arm, after achieving stable disease (SD) following four doses of tremelimumab followed by durvalumab monotherapy, the patient progressed at week 43, was retreated with tremelimumab, and was alive at week 67 at the time of data cut-off. Another patient with unknown PD-L1 and microsatellite status had an unconfirmed PR at week 18 in the monotherapy arm, and although their disease had progressed by week 24, the patient was alive at week 65. There were about 12% of patients with PD-L1 expression scores ≥ 25 in each arm, but there were not enough responders in this study to establish associations between biomarkers and clinical outcomes. The authors did report that out of 12 patients with SD, nine had tumors evaluable for PD-L1 expression, and all of these had low/negative PD-L1 status. While the objective response in the study was limited to one MSI-H patient, the observation that other patients may have still benefited by way of longer disease control highlights the need to identify biomarkers that predict benefit.

### 2.4. ICI Combined with Chemotherapy in PDAC

Our current SOC for advanced PDAC patients involves treatment with front-line chemotherapy [52]. FOLFIRINOX and combination gemcitabine plus nab-paclitaxel offer ORRs of 31.6% and 23%, respectively, while ORRs of single-agent gemcitabine or fluoropyrimidines used in patients intolerant of combination therapy range from 5–10% [2,3,62]. Front-line combination and single-agent chemotherapy results in mOS ranging from 8–11 months and six to seven months, respectively. Recent second-line chemotherapy regimens like 5-FU combined with liposomal irinotecan result in an mOS of 6.2 months [63]. Preclinical data suggest that chemotherapy-induced apoptosis may increase the immunogenicity of tumors through enhanced antigen presentation, T-cell reactivity, and T-cell tumor infiltration [64,65,66]. These findings support testing chemotherapy and ICI together in PDAC in the hope of enhancing the immune anti-tumor effect.

In two phase 1 studies in untreated advanced PDAC patients, CTLA-4 inhibitors combined with gemcitabine were evaluated. Aglietta et al. evaluated tremelimumab with gemcitabine and reported a 7.1% ORR with an mOS of 7.4 months [67]. Kalyan et al. studied ipilimumab with gemcitabine and reported a 12.5% ORR (two out of 16 patients with PR only), 43% disease control rate (DCR defined as CR, PR, and SD combined) with two PRs and five SDs, 2.5-month mPFS, and 8.5-month mOS [68]. Another phase 1 dose escalation and expansion study, which included mostly pretreated advanced PDAC patients (67% had received one prior line of therapy), studied ipilimumab with gemcitabine and reported an ORR of 14% (all PR) and an SD rate of 33% with a 47% DCR [69]. The mDOR was 11 months for the patients with PRs and 2.37 months for those with SD. Collectively, these authors concluded that combining CTLA-4 inhibitors with gemcitabine was safe and tolerable. Unfortunately, the responses and clinical outcomes reported were not significantly improved with CTLA-4 inhibitors added to gemcitabine when compared to historical controls of gemcitabine monotherapy. Correlative biomarker studies to differentiate responders from non-responders were unavailable due to insufficient numbers of samples.

ICI have also been combined with our current SOC combination chemotherapy regimens. In the PembroPlus phase 1/2 study, pembrolizumab combined with gemcitabine and nab-paclitaxel resulted in a 27% ORR (3 out of 11 patients with PR) in treatment-naïve mPDAC patients and 0% ORR in previously treated patients [70]. The primary endpoint of > 15% CR was not met. However, the patients with PR remained on treatment for 8-15 months. Ultimately, the authors concluded that the combination was safe in treatment-naïve patients, and efficacy was slightly improved over what has historically been reported with gemcitabine plus nab-paclitaxel. Unfortunately, in another phase 1 study by Wainberg et al. in untreated advanced PDAC patients, adding nivolumab (anti-PD-1) to gemcitabine plus nab-paclitaxel led to an 18% ORR, mPFS of 5.5 months, and mOS of 9.9 months, which was not an improvement on previously reported outcomes with combination chemotherapy alone [54]. In exploratory analyses, PFS and OS were not statistically significantly different based on tumor PD-L1 cut-offs. However, **o**n-treatment peak CD8+ and CD4+ T-cell numbers in the peripheral blood were higher in clinical responders (*p =* 0.03), and mPFS was longer in patients with higher versus lower peak on-treatment CD8+ T-cell levels. These findings, although exploratory, highlight low circulating CD8+ T-cell levels as a potential mechanism of ICI resistance.

Renouf et al. reported on a randomized phase 2 trial where 180 mPDAC patients were randomized to front-line treatment with gemcitabine plus nab-paclitaxel combined with durvalumab and tremelimumab or gemcitabine plus nab-paclitaxel without ICI [44]. Adding dual ICI to chemotherapy unfortunately did not improve ORR (30.3% with ICI vs. 23.0% without ICI; *p =* 0.096), DCR (70.6% vs. 57.4%; *p =* 0.96), mPFS (5.5 months vs. 5.4 months; *p =* 0.91), or mOS (9.8 months vs. 8.8 months; *p =* 0.72) but did increase the rate of grade 3 lymphopenia (*p =* 0.02). Plasma biomarker analyses in patients treated with IO found trends for improved OS in patients with a plasma TMB greater than or equal to 9 compared to those with lower plasma TMB scores, highlighting the potential for a low mutational burden to predict ICI inefficacy [71]. Most recently, Ueno et al. reported on phase 2 data from 31 mPDAC patients treated with front-line mFOLFIRINOX combined with nivolumab [45]. Although this was a tolerable regimen, ORR (32.3%), mPFS (7.39 months), and mOS (13.4 months) were again not dramatically different from historical mFOLFIRINOX-treated controls [2]. Of note, a Chinese study recently reported promising response rates (45.2%) and 93.5% DCRs when combining a novel bi-specific antibody, KN046, targeting PD-1/PD-L1 and CTLA-4 pathways with chemotherapy in advanced PDAC patients. KN046 with chemotherapy is now moving into phase 3 evaluation (ENREACH-PDA-01) [46]. Efforts are ongoing to identify novel ICI and chemotherapy combinations like this with the hope of improving outcomes.

### 2.5. ICI Biomarkers in PDAC

Overall, ICI alone or in combination with chemotherapy has led to disappointing results with either no additional activity or limited improvement in responses and survival. Aside from MSI-H/dMMR status, which is a well-established positive biomarker, additional analyses are crucial in identifying subgroups that may, in fact, be benefiting from ICI. In the PembroPlus study, changes in tumor cell-free DNA copy number instability (CNI) were retrospectively studied in nine patients treated with chemotherapy and pembrolizumab, and greater reductions in CNI were associated with improved PFS and OS [70]. In the study by Wainberg et al., exploratory biomarker analyses did not demonstrate significant differences in mPFS or mOS with nivolumab plus chemotherapy when stratified by baseline tumor PD-L1 status < 1% and ≥1% or <5% and ≥5% [54]. Additional post-hoc peripheral T-cell studies demonstrated increased CD8+ and CD4+ T-cell proliferation on treatment. Higher peak on-treatment values were seen in patients with a clinical response (*p =* 0.03), and these values were associated with longer PFS (*p =* 0.04) compared to lower peak values. Tumor immunohistochemistry (IHC) studies of T-cell markers in baseline and on-treatment samples did not reveal significant differences. Serum cytokine studies also suggested that while baseline IFNγ-responsive markers were not different between responders and non-responders, on-treatment peak CXCL10 levels were numerically higher in responders compared to non-responders (459 vs. 265 pg/mL; *p =* 0.10). While not statistically significant, mPFS was longer in patients with higher peak CXCL10 and sIL2Rα compared to lower levels.

Biomarker analyses from the Renouf et al. study reported trends for improved OS with higher plasma TMB values (≥9 mut/Mb) [71]. However, this TMB cut-off was seen only in a small subgroup (4.6%) of patients. In fact, molecular studies with over 700 PDAC patients demonstrated overall low incidences of TMB-high scores, PD-L1 positivity, and MSI-H status [72]. While it appears TMB and TME immune signatures may be predictive, although still not perfect, and PD-L1 alone is not a good biomarker in PDAC, these conclusions are derived from small patient numbers in PDAC and not validated. CXCR4 expression, homologous recombination repair gene mutations, and enrichment of select neoantigens, among others, are emerging as promising proposed biomarkers [73,74,75]. Additional tissue and plasma studies are warranted to identify novel biomarkers and prospectively validate those that are identified.

### 2.6. Novel Combinations with ICI to Enhance Immunogenicity in PDAC

Studies combining ICI with novel therapeutics to improve upon the disappointing results seen with ICI monotherapy or ICI-chemotherapy combinations in MSS PDAC are emerging in the preclinical and clinical settings. For example, preclinical studies using human PDAC models demonstrated that simultaneously blocking the CXCR4 alpha chemokine receptor and PD-1 enhances CD8+ T-cell migration and cytotoxicity [55]. In the clinical COMBAT phase 2 trial, a CXCR5 antagonist, motixafortide, was studied in heavily pretreated patients with advanced PDAC who had progressed on at least one prior therapy (56.8% received the trial drug as 3rd line therapy or beyond). This trial had two patient cohorts, one receiving motixafortide with pembrolizumab and the other receiving motixafortide with pembrolizumab plus chemotherapy [38]. In the motixafortide and pembrolizumab cohort, there was an ORR of 3.4% (one PR), 34.5% DCR, and mOS of 3.3 months. For patients receiving this as second-line treatment, mOS was 7.5 months.

While the numbers themselves may be unimpressive, it is interesting to see similar mOS between this non-chemotherapy immune-based regimen in the second-line setting compared to the mOS of 6.2 mo in patients treated with the approved second-line chemotherapy regimen, 5-FU plus nanoliposomal irinotecan (nal-IRI) in the NAPOLI-1 trial [63]. Motixaforide combined with 5-FU and nal-IRI improved on 5-FU and nal-IRI alone in the NAPOLI-1 trial (ORR, 32% vs. 17%; DCR, 77% vs. 52%). The immunotherapy combination resulted in increased activated TILs and decreased MDSC in the TME when baseline and paired on-treatment tumor biopsies were evaluated. The authors suggested that the addition of a CXCR5 antagonist and pembrolizumab to chemotherapy may enhance the benefit, and future randomized trials are warranted.

PDAC is also characterized by dense fibrous stroma that has been proposed as another mechanism of ICI resistance as it limits immune infiltration [76]. PEGPH20 is a pegylated, human recombinant PH20 hyaluronidase that remodels stroma and improves cytotoxic T-cell tumor infiltration and drug delivery to tumors, as seen in preclinical studies. A phase 2 study combined PEGPH20 with pembrolizumab in hyaluronidase-high, refractory mPDAC patients, yielding a 0% ORR and 25% SD rate (lasting 2.2 months and nine months), which were disappointing results [40,77]. Although PFS was 1.5 months, the mOS of 7.2 months was encouraging for a non-chemotherapy regimen in a heavily pretreated mPDAC population. Translational biomarker studies are pending.

Another novel ICI combination example is that of durvalumab with guadecitabine (an immunomodulating DNA methylase transferase inhibitor that upregulates interferon pathways), which was studied in pretreated patients with advanced PDAC in a recent phase 1 trial [78]. DCR was 33%, and although there was only one PR out of 24 patients, this was in an MSS PDAC patient with a durable response lasting over 24 months. Biomarker studies are pending.

Recently, combining pembrolizumab with a long-acting interleukin (IL)-7, called NT-I7, in a phase 2 trial resulted in responses in two out of 26 MSS PDAC patients, with best tumor reductions of 100% and 72%, respectively, suggesting that IL-7 may help overcome primary ICI resistance [42]. Other early phase studies combining ICI with immunomodulatory agents, such as anti-ICOS IgG, vitamin D receptor agonists, CXCL12 inhibitors, and CD40 agonists, are underway, and some have demonstrated safety and early efficacy signals in PDAC [79,80,81,82] (Table 2).

In the quest to identify novel biomarkers, large analyses have suggested that tumors harboring mutations in DNA damage repair pathways, specifically nucleotide excision repair and homologous recombination repair (HRR), may be prone to higher mutational burden and have increased susceptibility to IO therapy [74]. In PDAC, a single-institution, retrospective analysis of five advanced, refractory PDAC patients with germline mutations in HRR genes (including *BRCA1*, *BRCA2*, *RAD51C*, and *RAD51D*) treated with nivolumab and ipilimumab revealed a CR and a PR among three evaluable patients [83]. A recent phase 1/2 trial targeted DNA damage response with a poly (ADP ribose) polymerase combined with nivolumab or ipilimumab in the maintenance setting in patients with stable disease after platinum therapy. This study demonstrated promising results and better outcomes when the PARP inhibitor was combined with ipilimumab (15.4% ORR and 59.6% 6-month PFS) compared to nivolumab (7.1% ORR and 20.6% six-month PFS). In addition, novel promising targets for pharmacologic inhibition, such as heat shock protein 90 and multi-kinases, have shown the potential to sensitize immune-cold PDACs to IO therapy in preclinical studies [84,85]. Early studies have also demonstrated that targeting CSF1R, the CCL2-CCR2 chemokine axis, and Bruton tyrosine kinases, among many other targets, may modulate the immune milieu and could theoretically enhance ICI efficacy [86,87,88].

### 2.7. ICI and Radiation in PDAC

Radiation has historically been used in the peri-operative and advanced setting for local control and symptom management in PDAC patients. The abscopal effect is a unique phenomenon seen across tumor types where non-target metastatic lesions may exhibit tumoral regression after treatment of a target lesion with radiation [89]. This is thought to be due to radiation-induced cell death and antigen exposure at a treatment site leading to T-cell priming and immune responses at distant sites. In PDAC, preclinical mouse models have demonstrated promising results in multi-focal tumors when combining radiation and IO [90]. In clinical studies, the results are variable.

Xie et al. conducted a phase 1 study enrolling mPDAC patients who had received at least one prior line of therapy into four cohorts, where treatment consisted of durvalumab with stereotactic body radiation treatment (SBRT) given at either 8 Gy or 25 Gy doses or durvalumab combined with tremelimumab with SBRT delivered at either 8 Gy or 25 Gy doses [91]. No dose-limiting toxicities were reported. Out of all patients, 74.6% received radiation to the primary pancreatic lesion, while the rest received radiation to metastatic lesions in the liver or peritoneum, and a response was measured in lesions not treated with radiation. Of 39 evaluable patients, the ORR was 2.6% (including one confirmed PR and one unconfirmed PR, both in MSS patients), and DCR was 41.0%. The mPFS was 2.0 months, and the mOS was 3.7 months, and while PFS was similar among the cohorts, mOS was higher in patients who received the higher radiation dose compared to those who received the lower radiation dose. Response was not associated with the location of radiation. Immune correlates from five patients with matched baseline, and post-treatment tumor samples showed an increase in infiltrating CD3+ and CD8+ T cells but did not demonstrate any association between this and responses to treatment. While the primary objective of safety and tolerability was met, there was only a modest benefit from this approach.

Parikh et al. conducted a single-arm phase 2 study including 25 MSS mPDAC patients who progressed on prior therapy and were treated with radiation plus nivolumab and ipilimumab [48]. Response was measured in lesions outside the radiation field. In per-protocol analyses, the ORR was 18%, DCR was 29%, mPFS was 2.7 months, and mOS was 6.1 months, although mOS was 11.7 months in those who achieved disease control versus 4.4 months in those who did not. In biomarker analyses from baseline, pre-radiation, and post-radiation tumor samples, all patients were TMB low (<10 mut/Mb); TMB did not change throughout treatment, and TMB and DNA damage and repair pathway gene mutations identified did not correlate to response. However, RNA analyses from pre-treatment samples suggested that those with higher NK cell numbers were associated with disease control and response. This study demonstrated proof-of-concept of ICI combined with radiation, demonstrating some activity in advanced PDAC and identifying baseline NK cell infiltration as a potential biomarker in ICI trials.

Another phase 2 study published in 2022 demonstrated 2.4% and 14.0% responses in heavily pretreated MSS mPDAC patients treated with SBRT (15 Gy) and nivolumab or SBRT combined with nivolumab and ipilimumab, respectively [47]. DCRs were 17.1% and 37.2%, respectively, and mPFS and mOS were numerically similar. Biomarker analyses demonstrated no correlation between PD-L1 expression and outcomes. However, lower serum IL-6, IL-8, and C-reactive protein while on treatment, but not at baseline, were associated with clinical benefit. While these studies demonstrate safety and clinical activity, future studies are needed to validate these approaches, elucidate the individual contributions of radiation and ICI therapies, validate biomarkers, and clarify the optimal radiation dose, type of IO therapeutic, and sequencing of therapy.

## 3. Adoptive Cellular Therapy

### 3.1. Introduction to Adoptive Cellular Therapy

Adoptive cellular therapies are alternative forms of immunotherapy more commonly used in hematologic malignancies but increasingly explored in solid tumors. T-cell therapies, using the adoptive transfer of genetically modified, tumor-targeting T cells, represent a promising therapeutic modality for some difficult cancers, including PDAC [92]. Genetic modification often includes two main approaches. T cells can be engineered to (1) express T-cell receptors (TCRs) that recognize tumor antigens in the context of human leukocyte antigen (HLA) or (2) express chimeric antigen receptors (CARs) that directly bind to cancer cell-surface proteins, carbohydrates, or glycolipids, which allow them to overcome the HLA down-regulation commonly seen in solid tumors [93]. 

CAR T cells are generated by collecting autologous T cells from a patient’s blood through leukapheresis, genetically engineering them to express CAR specific for a specific tumor antigen, expanding them, and then re-infusing them into the patient [94]. CARs are composed of an antibody single-chain variable fragment (scFv) conjugated to intracellular signaling domains containing a CD3 zeta chain and one or more costimulatory domains such as CD28 and CD137 [92]. With a CAR scFv, the T cells can directly recognize cancer antigens independent of MHC antigen presentation, and CAR-specific recognition and binding to tumor antigen drive CAR T-cell activation and T-cell mediated tumor death [95]. The first generation of CARs designed to contain CD3 zeta or FcRγ signaling domains were limited by the lack of costimulatory signaling. Second and third-generation CAR T cells incorporate additional cytoplasmic costimulatory domains like CD28, CD137, and OX40 and tend to persist longer than first-generation models [92].

While studies in hematologic malignancies have shown that lymphodepletion with pre-conditioning regimens prior to CAR T-cell therapy improves the efficacy of CAR T cells, the role of pre-conditioning in solid tumors is less well established [96]. Lymphodepletion reduces the number of native T-cells in a patient, allowing room for infused CAR T-cells to utilize available cytokines, engraft, and expand [97]. Lymphodepletion also eliminates some immunosuppressive cells like TAMs, MDSC, and T-regs in the TME. The most common chemotherapies used in CAR T-cell trials are cyclophosphamide alone or in combination with fludarabine (Cy/Flu), but these are not traditionally used to treat PDAC directly. Whether these or cytotoxic regimens commonly used against PDAC (gemcitabine or fluoropyrimidine-based therapies), which have previously demonstrated the potential to modify the TME through immunogenic cell death, local T-cell infiltration, and T-reg and MDSC eradication, add benefit to CAR T-cell therapy remains under investigation [98].

### 3.2. CAR T-Cell Therapy in PDAC

The target antigens in multiple early-phase CAR T-cell clinical trials for pancreatic cancer include mesothelin (MSLN), prostate stem cell antigen (PSCA), carcinoembryonic antigen (CEA), HER2, MUC1, and CD133. Mesothelin, a cell-surface antigen expressed in about 80–85% of PDAC, has been the most common CAR T-cell target in PDAC [99]. Unfortunately, in a phase 1 study including five PDAC patients treated with mesothelin-targeting CAR T cells, two patients experienced SD that persisted at two and three months follow-up, while the other three had POD as the best response [100]. While post-treatment tumor biopsies revealed the presence of CAR T cells, the numbers were quite low, and CAR T persistence was transient in the peripheral blood, potentially explaining the general lack of response. In addition, the presence of mesothelin was not required for screening and was detected in only three of the patient’s tumors. Another phase 1 study included six treatment-refractory metastatic PDAC patients who were treated with CAR T cells three times per week for three weeks [101]. While the treatment was safe and feasible (no dose-limiting toxicity, cytokine release syndrome, or neurologic toxicities were observed), the best response was SD, lasting 3.8 and 5.4 months in two patients. Interestingly, positron-emission tomography showed dramatic metabolic activity reduction in the liver lesion of one patient, highlighting the potential anti-tumor activity. Several other trials targeting mesothelin are underway (Table 3).

In a phase 1 trial including seven treatment-refractory, advanced PDAC patients, CAR T cells targeting CD133 (a marker expressed by cancer stem cells) resulted in two PR, three SD (one of which was over 10 months), and two POD [102]. Another phase 1 study enrolling EGFR-positive PDAC patients treated with CAR T cells targeting EGFR demonstrated a DCR of 85.7% involving four PRs lasting two to four months out of 14 evaluable patients. Despite the impressive DCR, mPFS was only 3.0 months, and mOS was 4.9 months [103]. Furthermore, multiple patients experienced Grade 3–4 hematologic, skin, gastrointestinal, and pulmonary toxicities, likely reflecting the off-target effects of therapy, as many normal epithelial cells express EGFR. Another phase 1 trial targeting HER2 with CART-HER2 cells yielded stable disease in two of two HER2-positive PDAC patients; their PFS was 5.3 and 8.3 months [104]. Again, while a pre-conditioning regimen led to T-cell expansion, CAR T-cell persistence at therapeutic levels was limited.

Numerous ongoing phase 1 trials are attempting to expand on the targets discussed above and evaluate other targets such as CEA, claudin 18.2, MUC1, and PSCA (Table 3). Although some responses have been noted in small numbers of patients, ongoing research is needed to construct CAR T cells that improve survival and minimize off-target toxicities.

### 3.3. Limitations and Future Directions of CAR T-Cell Therapy in PDAC

Although CAR T-cell therapy has advanced within the last decade, its application as a treatment for PDAC remains in its infancy, albeit with promising potential. Major obstacles for CAR T-cell therapies in PDAC include immunosuppressive TMEs and dense fibrous stroma that limit endogenous immune infiltration and function. Combining CAR T cells targeting immunosuppressive cells with tumor-targeting CAR T cells could potentially overcome the TME barrier. A phase 1 pilot study with three patients evaluated the safety and feasibility of this type of dual CAR T-cell therapy approach [105]. Patients were treated with one infusion of anti-MSLN immunoreceptor CAR T-cells designed to target PDAC and a separate infusion of anti-CD19 immunoreceptors CAR T- cells designed to target and deplete CD19 B-cells. These B-cells were hypothesized to limit in vivo persistence of CAR T cells and decrease T-cell immunosurveillance. Although B-cells were depleted with therapy, this, unfortunately, did not significantly prolong CAR T-cell persistence or improve clinical activity. However, studies using CAR T cells to target other suppressors, like TAMs, have shown promising outcomes in pre-clinical studies and remain an area of ongoing research [106].

Additionally, although second-generation CAR T cells are endowed with simultaneous co-stimulation mechanisms that overcome their natural tendency toward anergy, the upregulation of co-inhibitor receptors in the TME, such as PD-1 or CTLA-4, has limited the efficacy of CAR T cells in preclinical studies [107]. Combining CAR-T-cell therapy with ICI may potentially overcome this barrier. There are limited clinical trials evaluating this approach today, and results have been conflicting when reported in patients with other cancer types [108,109]. However, innovative strategies like constructing CAR T cells that target PD-1 and PD-L1 have led to dramatic results in xenograft and orthotopic tumor models to date [110]. Preclinical studies have also shown enhanced efficacy when combining CAR T cells with other therapies like oncolytic adenoviruses expressing pro-inflammatory cytokines that modulate the PDAC TME [111].

In addition, many studies to date demonstrate that CAR T cells are limited by transient persistence after expansion in the peripheral blood [100,104,112]. Although the mechanism for this is ill-defined, antibodies against CARs have been detected previously, and efforts to deplete patients of lymphocytes and T cells with pre-conditioning regimens or simultaneously targeting B-cells with CAR T cells have failed to improve CAR T-cell persistence [100]. Numerous trials using different CAR constructs (human and murine variable fragments) and pre-conditioning approaches are underway (Table 3).

Another major challenge is identifying targetable markers that are enriched in most PDAC but not normal tissue to enhance anti-tumor activity and minimize off-target toxicity. This is an ongoing effort, and investigators have recently identified novel targets like CEACAM7, an extracellular surface protein present on many PDACs and restricted to pancreatic ductal cells and epithelial colon cells, with promising preclinical data [113]. Targeting neoantigens, using multiple antigens simultaneously, and using stromal components are other proposed methods moving forward [75,114]. For example, Zhang et al. designed novel CAR T cells, termed dual-receptor CAR T cells, that had two CARs specific for CEA and MSLN and required dual antigen-receptor interaction to activate T-cells [115]. There was significant anti-tumor activity against pancreatic cancer cell lines and xenograft tumor models expressing both CEA and MSLN, and there was a lack of T-cell activation in the presence of just one antigen. These results were promising for enhancing the cytotoxic activity of CAR T cells, specifically for tumor cells, while sparing off-tumor normal tissue targets. Early studies have also demonstrated the feasibility of improving local drug delivery to metastases through hepatic artery infusion of targeted CAR T cells [116]. Improving immunotherapy efficacy by combing T-cell therapies with novel immune-enhancing agents such as ICI, T-reg-depleting therapies, costimulatory molecules like rimiducid, peroxisome proliferator-activated receptor gamma ligands, tyrosine kinase inhibitors, and other drugs that regulate inflammation may also be worth further investigation [117,118,119,120,121].

In addition, although data are not extensive, other adoptive cellular approaches utilizing alternative strategies like CAR-NK cells or TIL therapy are increasingly being explored [122,123]. Most recently, a combination phase 2 protocol included metronomic low-dose chemoradiation, cytokine-induced NK and T-cell activation via an IL-15 cytokine fusion protein, and a novel PD-L1-targeted NK cell infusion in a heavily pretreated, refractory population (over half of the patients were on at least fourth line therapy). An mOS of 5.8 months in all patients and mOS of 6.3 months, specifically in patients on third-line therapy, were observed [124]. These survival data were promising when compared to the mOS of three months after third-line therapy in historical controls.

## 4. Vaccine Therapy

### 4.1. Vaccine Therapies in PDAC

Vaccine therapy, another form of IO, works by exposing the immune system to cancer-associated antigens to prime T cells and boosting an anti-cancer immune response. Vaccines can deliver cancer-specific antigens in the form of peptides, whole tumor cells, APCs like dendritic cells (DC), DNA, and micro-RNA (mRNA), for example. This strategy has been widely studied with variable outcomes (Table 4).

DC vaccines present antigens to CD4+ and CD8+ T cells and secrete cytokines such as IL-15, IL-12, IFN-γ, and TNF, thereby promoting the activation of cytotoxic CD8+ T cells [151]. In a pilot phase 1 trial by Rong et al., an intradermal delivery of MUC1-peptide-pulsed DCs in advanced PDAC patients led to no clinical responses across seven patients, but the intervention was tolerable and showed signals of increasing IFN-γ and granzyme B production in peripheral blood mononuclear cells [152]. A similar 0% ORR has been reported in other studies of DC pulsed with various peptides [153,154]. Other studies using DC vaccines combined with chemotherapy or additional immunogenic agents like cytokine-induced killer cell-based therapies have resulted in modest 14–20% PRs in small numbers of patients [155,156]. However, throughout most studies, regardless of clinical responses, vaccine therapies seem to induce cytotoxic lymphocytes and enhance cytotoxic cytokine signaling, thereby suggesting that DC approaches may enhance immunity but require alternative combination therapeutics to improve clinical efficacy.

Peptide vaccines, or vaccines composed of amino acid sequences representing epitopes of cancer-specific antigens, may also induce an adaptive immune response. Multiple studies using RAS, MUC1, GV1001 (telomerase), survivin, G17DT, vascular endothelial growth factor receptor (VEGFR), CEA, WT1, and personalized neoantigen vaccines, among others, have been reported (Table 4). Many early phase 1/2 studies using peptide vaccines without combination therapy in advanced disease were tolerable but resulted in minimal to no clinical responses or improvements in survival [125,126,131,157,158,159]. However, SD was achieved in about 17–60% of patients, and trends for improved survival were often reported amongst those patients who experienced T-cell/immune responses after vaccination.

Targeting gastrin, a gastrointestinal peptide that promotes epithelial-mesenchymal transition and metastases in PDAC through the β-catenin pathway, has been of interest based on data suggesting that anti-gastrin therapies downregulate desmoplastic fibrosis in TME and induce T-cell activation in preclinical models [160,161]. In a phase 3 trial by Gilliam et al., advanced PDAC patients were randomized to treatment with G17DT (an immunogen that blocks gastrin-mediated growth) versus placebo, and mOS was improved by the vaccine, although modestly (151 days versus 82 days; *p =* 0.03), suggesting some potential anti-tumor benefit [136]. In addition, those with anti-G17DT immune responses tended to have longer survival. When combined with chemotherapy in randomized phase 2 and 3 studies, peptide vaccines such as those with survivin, G17DT, and VEGFR did not improve PFS or OS when compared to chemotherapy alone in locally advanced/advanced PDAC patients [132,133,134,135,137]. Although multiple studies have demonstrated that immune responses may be induced with vaccination, and these may be associated with clinical responses or survival in the adjuvant or advanced setting, these data have limitations. They are from small numbers of patients in early-phase trials lacking a randomized comparator arm or subgroup analyses in randomized trials. We currently lack strong randomized data indicating significant survival benefits from incorporating peptide vaccines in the advanced or adjuvant setting.

GVAX vaccine, or allogenic, whole pancreatic cancer cells modified to express granulocyte-macrophage colony-stimulating factor (GM-CSF), sometimes given with cyclophosphamide to deplete Tregs, has been shown to induce T-cell infiltration in the PDAC TME [141,162]. In the adjuvant setting, GVAX in a phase 1 trial was safe, induced immune responses, and three out of 14 patients who experienced a delayed-type hypersensitivity reaction remained disease-free at 25 months after diagnosis [163]. In another adjuvant single-arm, phase 2 study, GVAX followed by 5-FU based chemoradiation resulted in a one-year DFS rate of 67.5% and one-year OS rate of 85%, and enhanced T-cell responses were associated with longer DFS [142]. A recent multi-arm, phase 2 trial using a neoadjuvant and adjuvant vaccine with and without ICI therapy reported no mDFS benefit when adding nivolumab to GVAX (16.23 months vs. 14.82 months; *p =* 0.96) and only marginally significantly improved mDFS when nivolumab and a CD137 agonist was added to GVAX (not reached vs. 14.82 months; *p =* 0.097) [145]. Another phase 3 randomized trial comparing neoadjuvant chemotherapy plus chemoradiotherapy plus algenpantucel-L (allogenic pancreas cancer cells expressing murine ɑ(1,3)GT gene) to chemotherapy and chemoradiotherapy alone in borderline and locally advanced PDAC demonstrated no PFS or OS advantage with the addition of IO therapy [146].

In advanced disease, GVAX plus cyclophosphamide combined with live-attenuated, mesothelin-expressing *Listeria monocytogenes* (CRS-207) was compared to GVAX plus cyclophosphamide in a randomized trial, and although both arms had 0% ORR and no difference in PFS, there was an mOS advantage with adding CRS-207 (6.1 mo vs. 3.9 mo; p<0.02) [147]. However, in a subsequent phase 2 trial randomizing patients to GVAX plus cyclophosphamide plus CRS-207 vs. CRS-207 alone vs. single-agent physician choice, chemotherapy showed no survival advantage with the combination approach or CRS-207 alone over chemotherapy [148].

### 4.2. Limitations and Future Directions of Vaccine Therapies in PDAC

Most vaccine therapy clinical trials to date in PDAC have not demonstrated dramatic improvements in survival. However, most of these studies do demonstrate signals of increased cancer antigen-specific T-cell responses after vaccination. In addition, as discussed above, these responses are sometimes associated with longer survival and clinical responses. This suggests that our current approaches may be inducing immune activation, but other therapeutic combination partners or antigen targets based on preclinical studies should be considered to augment these responses to be clinically meaningful. For example, studies in murine models suggest GVAX upregulates PD-L1 membrane expression on tumor cells, and combining GVAX with ICI improves survival [164]. Other preclinical studies suggest synergy in IL-3 production and activated CD4+ T-cell tumor infiltration when combining DC vaccines with anti-CTLA-4 therapy [165]. Multiple phase 1 clinical trials of novel vaccine (DC, peptide, and other forms) combinations, adjuvant stabilizing solutions, antigen targets, personalized approaches, and combinations with other IO therapies, including ICI across various clinical settings (high-risk patients, adjuvant, advanced, and minimal residual disease) are underway (NCT03592888, NCT05013216, NCT04853017, NCT04117087, NCT02600949, NCT03767582, NCT04799431).

## 5. Conclusions

Collectively, the studies utilizing IO therapies, especially ICI, to date have demonstrated minimal improvements in PDAC patient outcomes. Tumor genomic and immune-signature analyses over the years have begun to elucidate the inherent immune-cold nature of PDAC, offering some explanation for PDAC’s IO refractoriness. However, we also recognize that subsets of patients exist who do benefit from IO, but further studies are needed to identify what distinguishes these patients. Ongoing translational analyses in these trials are crucial to identify predictive biomarkers and mechanisms for primary and secondary IO resistances, which may facilitate novel drug discovery to overcome these barriers. Major research efforts are necessary to improve outcomes for the PDAC patient population who still rely primarily on chemotherapy, face dismal survival rates, and have not yet experienced the potential dramatic benefits with IO seen in other malignancies.

## Figures and Tables

**Table 1:** FDA Approved Immune Checkpoint Inhibitors.

Immune Checkpoint Inhibitor	Monoclonal Antibody Target
Pembrolizumab	PD-1
Nivolumab	PD-1
Avelumab	PD-1
Cemiplimab	PD-1
Dostarlimab-gxly	PD-1
Atezolizumab	PD-L1
Durvalumab	PD-L1
Ipilimumab	CTLA4
Relatlimab	LAG-3

Glossary: CTLA4: cytotoxic T-lymphocyte-associated antigen 4; LAG-3: lymphocyte-activation gene 3; PD-1: programmed cell death protein 1; PD-L1: programmed cell death protein ligand 1.

**Table 2:** Examples of Resulted Phase 2 ICI Clinical Trials in Advanced PDAC.

Study Reference (Phase)	Population (Line of Treatment)	Intervention	ORR	DCR	mPFS (mo)	mOS (mo)
ICI MONOTHERAPY						
[33] (2)	mPDAC/LAPC n = 27	ipi	0% (2 pts with minor response)	-	-	No benefit
[34] (2)	mPDAC (2nd+) n = 20	treme	0% (18/20 POD)	0%	-	4.0
KEYNOTE-158 [35] (2)	Advanced PDAC (2nd) n = 22 (100% MSI-H/dMMR)	pembro	18.2% (1 CR, 3 PR, mDOR 13.4 mo [8.1–16+ mo])	-	2.1	4.0
**ICI + OTHER TARGETED THERAPY**						
[36] (1/2)	Advanced PDAC (2nd+) n = 49	durvalumab + ibrutinib (Bruton tyrosine kinase inhibitor)	2% (1 PR, DOR 10 mo)	-	2	4.0
[37] (2)	mPDAC (2nd) n = 65	Randomized to:		At 3 mo:		
		treme + durva	3.1% (1 PR in an MSI-H pt, DOR 55 weeks)	9.4%	1.5	3.1
		durva	0%	6.1%	1.5	3.6
COMBAT [38] (2)		Cohort 1				
	mPDAC (2nd +) n = 37	pembro + motixafortide (CXCR4 antagonist)	3.4% (1 PR)	34.5% (9 SD, 1 PR)	-	3.3 (ITT), 7.5 (2nd line only)
		Cohort 2				
	n = 22	pembro + chemo + motixafortide	32% (7 PR)	77% (10 SD, 7 PR)		
[39] (2)	LAPC/m PDAC (2nd +) n = 77	acalabrutinib (Bruton tyrosine kinase inhibitor)	0%	14.3%	1.4	3.6
		pembro + acalabrutinib	7.9% (all PR)	21.1% (mDOR 3 mo)	1.4	3.8
PCRT16-001 [40] (2)	Hyaluronan-high, mPDAC (3rd +) n = 8	pembro + PEGPH20 (human recombinant PH20 hyaluronidase)	0%	25% (2 SD [DOR 2.2 and 9 mo, each])	1.5	7.2
[41] (1/2)	Advanced PDAC (without progression >4 mo on platinum) n = 91	nivo + niraparib (PARP inhibitor)	7.1%	-	1.9	14.0
		ipi + niraparib	15.4%	-	8.1	17.3
[42] (2)	Advanced PDAC (relapsed/refractory) n = 32	pembro + NT-17 (long-acting interleukin-7)	8% (2/26, best tumor reduction 100% and 72%, respectively)	DOR >1.35 mo and 6.64 mo respectively	-	-
[43] (2)	Advanced PDAC (1st) n = 105	nivo + chemo	-	74% (mDOR 7.4 mo)	6.4	16.7
		sotigalimab (anti-CD40) + chemo	33%	78% (mDOR 5.6 mo)	7.3	11.4
		nivo + sotigalimab + chemo	31%	69% (mDOR 7.9 mo)	6.7	10.1
**ICI + CHEMOTHERAPY**						
[44] (2)	mPDAC (1st) n = 180	Randomized (2:1) to:				
		durva + treme + gemcitabine + nab-paclitaxel	30.3%	70.6%	5.5	9.8
		gemcitabine + nab-paclitaxel	23.0%	57.4%	5.4	8.8
[45] (2)	Advanced PDAC (1st) n = 31	nivo + mFOLFIRINOX	32.3% (all PR, mDOR 7.36 mo [3.5–20.1+ mo])	-	7.39	13.4
[46] (2)	Advanced PDAC n = 53	KN046 (bispecific antibody targeting PD-1/PD-L1 and CTLA-4) + gemcitabine + nab-paclitaxel	45.2%	93.5%	-	-
	Advanced PDAC n = 53	KN046 (bispecific antibody targeting PD-1/PD-L1 and CTLA-4) + gemcitabine + nab-paclitaxel	45.2%	93.5%	-	-
**ICI + LOCAL THERAPY**						
CheckPAC [47] (2)	Refractory mPDAC (2nd line +) n = 84	Randomized to:				
		SBRT (15 Gy) + nivo (100% pMMR)	2.4% (0% CR, 2.4% PR, 14.6% SD, 68.3% POD), mDOR 4.6 mo	17.1%	1.7 (1.6–1.8)	3.8 (3.1–5.8)
		SBRT (15 Gy) + nivo/ipi (97.7% pMMR, 2.3% unknown)	14.0% (0% CR, 14% PR, 23.3% SD, 53.5% POD), mDOR 5.4 mo (4.2-NR)	37.2%	1.6 (1.6–2.8)	3.8 (2.8–6.5)
[48] (2)	mPDAC N = 25 (100% MSS)	Radiation + nivo + ipi	ITT: 12%	20%	2.5	4.2
			Per-protocol: 18%	29%	2.7	6.1

Glossary: 5-FU: 5-fluorouracil; atezo: atezolizumab; CR: complete response; CRP: C reactive protein; DCR; disease control rate; DOR: duration of response; durva: durvalumab EGFR: epidermal growth factor receptor; FOLFOX: 5-Fluorouracil, oxaliplatin, leucovorin; ICI; immune checkpoint blockade; ipi: ipilimumab; ITT; intention to treat; LAPC: locally advanced pancreatic cancer; mets: metastases; mPFS: median progression-free survival; mOS: median overall survival; MSS: microsatellite stable; MSI-H: microsatellite instable-high; MTD: maximum tolerated dose; mut/Mb; mutations per megabase; nivo: nivolumab; NK: natural killer; NR: not reached; ORR: overall response rate; PARP: poly (ADP ribose) polymerase; PDAC: pancreatic ductal adenocarcinoma; pembro: pembrolizumab; POD: progression of disease; PR: partial response; SBRT: stereotactic body radiation therapy; TMB: tumor mutational burden; treme: tremelimumab; Tx: treatment; VEGFR: vascular endothelial growth factor receptor.

**Table 3:** Ongoing CAR Trials in PDAC.

Target	Phase	CAR Cells (Additional Therapy)	Institution	Study Identifier
Claudin 18.2	I	T-cells	PLA General Hospital, Beijing, China	NCT05275062
	I	T-cells	University of Southern California, Los Angeles, CA; University Of California San Diego, San Diego, CA; Moffit Cancer Center, Tampa, FL; Mayo Cancer Hospital, Rochester, MN; Baylor Charles Sammons Cancer Center, Dallas, TX; MD Anderson Cancer Center, Houston, TX	NCT04404595
	I/II	T-cells	Beijing Cancer Hospital, Beijing, China; Henan Tumor Hospital, Zhengzhou, China; Ruijin Hospital, Shanghai, China	NCT04581473
	I	T-cells	The Affiliated Hospital of Xuzhou Medical University, Jiangsu, China	NCT04966143
	I	T-cells	Shenzhen Luohu Hospital, Shenzhen, China	NCT05277987
	I	T-cells	Peking University Cancer Hospital, Beijing, China	NCT05393986
	I	T-cells	Peking University Cancer Hospital, Beijing, China	NCT03874897
PSCS	I/II	T-cells (Rimiducid)	Moffit Cancer Center, Tampa, FL; Emory Winship Cancer Institute, Atlanta, GA; John Theurer Cancer Center, Hackensack University Medical Center, Hackensack, NJ; Columbia University Medical Center, New York, NY; Baylor Charles Sammons Cancer Center, Dallas, TX; MD Anderson Cancer Center, Houston, TX	NCT02744287
HER2	I	T-cells (intra-tumoral CAdVEC oncolytic adenovirus injection)	Baylor St Luke’s Medical Center, Houston, TX	NCT03740256
	I	Macrophages	City of Hope National Medical Center, Duarte, CA; University of North Carolina Lineberger Comprehensive Cancer Center, Chapel Hill, NC; Abramson Cancer Center, Philadelphia, PA; Sarah Cannon Research Institute, Nashville, TN; MD Anderson Cancer Center, Houston, TX	NCT04660929
ROR2	I	T-cells (cyclophosphamide and fludarabine lymphodepletion)	Zhongshan Hospital, Shanghai, China	NCT03960060
GUCYC	I	T-cells	Beijing Cancer Hospital, Beijing, China	NCT05287165
	I/II	T-cells	Chingqing University Cancer Hospital, Chongqing, China	NCT04348643
	I	T-cells	Zhejiang University, Zhejiang China	NCT05396300
EpCAM	I/II	T-cells	Chengdu Medical College, Chendu, China	NCT03013712
	I	T-cells	Zhejiang University, Hangzhou, China	NCT05028933
EpCAM,	NA	T-cells (anti-TM4SF1 CAR T-cells)	Institution for National Drug Clinical Trials, Tangdu Hospital, Tangdu, China	NCT04151186
CD70	I/II	T-cells (non-myeloablative, lymphodepleting regimen + aldesleukin)	National Institutes of Health, Bethesda, MD	NCT02830724
CD276	I/II	T-cells	Shenzhen University General Hospital, Guangdong, China	NCT05143151
Mesothelin	I/II	T-cells	Shanghai Tumor Hospital, Shanghai, China	NCT02959151
	I	T-cells	First Affiliated Hospital of Wenzhou Medical University, Wenzhou, China	NCT03497819
	NA	T-cells (cyclophosphamide lymphodepletion)	Nanjing First Hospital, Nanjing, China	NCT03638193
	I/II	T-cells	First Affiliated Hospital of Zhengzhou Medical University, Zhengzhou, China	NCT03638206
	I	T-cells	PLA General Hospital, Beijing, China	NCT02580747
	I		Renji Hospital, Shanghai, China	NCT02706782
	I	T-cells	University of Pennsylvania, Philadelphia, PA	NCT03323944
	I	T-cells (VCN-01 oncolytic adenovirus)	University of Pennsylvania, Philadelphia, PA	NCT05057715
Mesothelin/PSCA/CEA/HER 2/MUC1/EGFR	I	T-cells	Harbin Medical University, Harbin, China	NCT03267173
MUC1	I/II	Natural killer cells	The First People Hospital of Hefei, Hefei, China	NCT02839954
	I/II	T-cells	The First People Hospital of Hefei, Hefei, China	NCT02587689
	I	T-cells (Rimiducid)	Sarah Cannon Research Institute, Denver, CO; NEXT Oncology, San Antonio, TX	NCT05239143

**Table 4:** Examples of Phase 2 or 3 Trials Using Vaccine Strategies in PDAC.

Study Citation (Phase)	Treatment Setting	Intervention	ORR	mPFS/ mDFS	mOS	Comments
PEPTIDES						
[125] (1/2)	-Advanced	-RAS-loaded APC	-0% (60% SD)	-	10.5 mo (if T-cell response) vs. 4.5 mo (no T-cell response)	-40% T-cell responses
[126] (1/2)	-Adjuvant	-RAS + GM-CSF	-	-4/10 pts remained NED at 22–39 mo	-25.6 mo	-
	-Advanced		-0% (32% SD)		-NA	-mDOR in responders 10.2 mo
[127] (2)	-Adjuvant	-RAS + DETOX adjuvant	-	- 11–64+ mo	-20–47+ mo	-60% immune responses, all experienced ongoing DFS. -Those without immune response had POD.
[128] (2)	-Adjuvant	-RAS + GM-CSF	-	-	-27.5 mo (all) -28 mo (immune responders)	-85% immune responses (3 pts had memory response up to 9 years) -10-year survival 20% vs. 0% in vaccinated vs. non-vaccinated cohort
[129] (1/2)	-Adjuvant	-TG01 (KRAS) + GM-CSF + gemcitabine	-	-13.9 mo	-33.1 mo	-92% immune response -Favorable DFS and OS compared to historic adjuvant controls with gemcitabine
[130] (2)	-Adjuvant	Randomized to:				
		KRAS (expressed on inactivated yeast) + gemcitabine		-	In R1 group: -523.5 days	-159 day improvement in OS in R1 pts (*p* = 0.872)
		-placebo + gemcitabine		-	-443.5 days	-Increased immune responders with the vaccine (40% vs. 8%, *p* = 0.062)
[131] (1/2)	-Advanced	-GV1001 (telomerase) + GM-CSF	-	-	-7.2 mo for immune responders vs. 2.9 mo for non-responders (*p* = 0.001)	63% immune response
[132] (3)	Advanced, treatment-naïve	Randomized to:				
		-gemcitabine	-	-3.7 mo	-7.3 mo	-PFS HR 0.5; 95% CI 0.4–0.7 -OS HR 0.8; 95% CI 0.6–1.0 -Vaccine did not improve OS
		-GV1001 + GM-CSF + concurrent gemcitabine if POD	-	-1.9 mo	-5.9 mo	
[133] (3)	-Advanced, treatment-naïve	Randomized to:				
		-chemotherapy (gemcitabine + capecitabine)	14.03% collectively	-	-7.9 mo	-Vaccine did not improve survival
		-chemotherapy→ GV1001 + GM-CSF			-6.9 mo	
		-chemotherapy + concurrent GV1001 + GM-CSF			-8.4 mo	
[134] (2)	-Advanced, treatment-refractory	Randomized to:				
		-survivin + IFNβ + Freund’s adjuvant	-no difference in DCRs between groups	-no difference between groups	-no difference between groups	-Vaccine did not improve PFS but did show an immunologic reaction
		-survivin + Freund’s adjuvant				
		-placebo				
[135] (2/3)	-Advanced	Randomized to:				
		-G17DT + gemcitabine	-	-similar PFS 3.9 mo	-5.8 mo	-Vaccine did not improve PFS/OS
		-placebo + gemcitabine	-	-similar PFS 3.9 mo	-6.6 mo	
[136] (3)	-Advanced	Randomized to:				-mOS was improved with the vaccine (*p* = 0.03) -73.8% with anti-G17DT responses, associated with longer survival
		-G17DT	-	-	-151 days	
		-placebo	-	-	-82 days	
[137] (2/3)	-Locally advanced, advanced, treatment-naïve	Randomized to:				
		-VEGF2 + Freund’s adjuvant + gemcitabine	-59.6% DCR	-3.71 mo	-8.36 mo	-Vaccine did not improve PFS/OS
		-placebo + gemcitabine	-60.4% DCR	-3.75 mo *p* = 0.313	-8.54 mo *p* = 0.918	
[138] (2)	-Advanced, treatment-naïve	-Personalized reactive peptides + Freund’s adjuvant + gemcitabine	-33% (all PR, 43% SD, 76% DCR)	-	-9 mo	56% immune responses (associated with improved survival)
[139] (2/3)	-Advanced, treatment-refractory	- KIF20A-66 + Freund’s adjuvant	-0% (72% SD, 72% DCR)	-56 days	-142 days	-27.6% objective tumor responses (but not by RECIST criteria) -1 pt with SD achieved CR over time -OS improved when compared to control, non-vaccinated group (*p* = 0.002)
[140] (2)	-Adjuvant	-VEGFR1, VEGFR2, KIF20A + gemcitabine	-	-15.8 mo	NR (18 mo follow-up)	-Survival was improved in KIF20A-expressing pts compared to non-expressors
**TUMOR CELLS**						
[141] (Pilot, feasibility)	-Advanced	Randomized to:				-Adding Cy induced more T-cell responses and was associated with longer ORR/OS
		-GVAX	-16.7% SD		-2.3 mo	
		-Cy + GVAX	-40% SD		-4.3 mo	
[142] (2)	-Advanced	-GVAX followed by 5-FU-based chemoradiation	-	- 17.3 mo	-24.8 mo	-Enhanced T-cell responses were associated with DFS
				-1-y DFS 67.4%	-1-y OS 85%	
[143] (2)	-Adjuvant	-Algenpantucel-L + gemcitabine + 5-FU-based chemoradiation	-	-12-mo DFS 62%	12-mo OS 86%	-Survival compares favorably to historical adjuvant data at the time
				-mDFS 14.1 mo	-mOS NR	
[144] (1/2)	-Adjuvant	-Pancreatic CSC vaccine	-	-	-	-CSC-specific immunity and lysis were higher post-vaccination -CSC-non-specific responses were also increased.
[145] (2)	-Resectable	Enrolled to neoadjuvant + adjuvant tx arms:				-No DFS benefit to adding nivolumab to GVAX alone (*p* = 0.96), and triplet was marginally significantly improved compared to GVAX alone (*p* = 0.097)
		-GVAX		-14.82 mo	-25.0 mo	
		-GVAX + nivolumab		-16.23 mo	-26.4 mo	
		-GVAX + nivolumab + CD137 agonist		-NR	-NR	
[146] (3)	-Borderline resectable or locally advanced	Randomized to neoadjuvant treatment arms:				HAPa immunotherapy did not improve PFS/OS
		-allogenic pancreas cancer cells expressing murine ɑ(1,3)GT gene (HAPa) + chemotherapy +chemoradiation	-	-12.4 mo	-14.3 mo	
		-chemotherapy + chemoradiation	-	-13.4 mo *p* = 0.59	-14.9 mo *p* = 0.98	
**TUMOR CELLS + BACTERIA**						
[147] (2)	-Advanced	Randomized to:				
		-Cy + GVAX + CRS-207 (live-attenuated mesothelin-expressing *Listeria monocytogenes*)	-0% (31% SD)	-No difference in PFS between arms	-6.1 mo	-First study to demonstrate a survival advantage with IO in PDAC
		-Cy/GVAX	-0% (24% SD)		-3.9 mo (HR 0.59, *p* < 0.02)	-Enhanced mesothelin-specific T-cell responses were associated with OS
[148] (2)	-Advanced, previously treated	Randomized to:				-Cy + GVAX + CRS-207 and CRS-207 monotherapy did not improve survival over chemotherapy
		-Cy + GVAX + CRS-207	-1.5% (1 PR, DCR 23.5%)	-2.3 mo	-3.7 mo	
		-CRS-207	-0% 13.8% (DCR 13.8%)	-2.1 mo	-5.4 mo	
		-Single-agent physician choice chemotherapy	-0% (DCR 11.6%)	-2.1 mo	-4.6 mo	
**BACTERIA VECTORS**						
[149] (2)	-Advanced	Randomized to:				-Subgroup analysis: metastatic subgroup (84%), OS improved from 4.4 to 7 mo with the addition of IMM-101 (*p* = 0.01)
		-IMM-101 (heat-killed *Mycobacterium obuense*) + gemcitabine	-10.7% (all PR)	-4.1 mo	-6.7 mo	
		-Gemcitabine	-2.9% (all PR) (*p* = 0.164)	-2.4 mo (*p* = 0.016)	-5.6 mo (*p* = 0.074)	
**VIRAL VECTORS**						
[150] (2)	-Advanced, treatment-naïve	Randomized to:				
		-Pelareorep (reovirus targeting RAS-activated tumors) + carboplatin + paclitaxel	-19% (all PR, 53% SD, 556% DCR)	-4.9 mo	-7.3 mo	-KRAS mutational status did not predict survival -Virus did not improve PFS/OS
		-19% (all PR, 49% SD, 59% DCR)	-19% (all PR, 49% SD, 59% DCR)	-5.2 mo (*p* = 0.6)	-8.8 mo (*p* = 0.68)	-Increased NK-cells and B-cells were associated with improved DCR

Glossary: AE: adverse event; APC: antigen presenting cell; cape; capecitabine; CEA: carcinoembroyonic antigen; CI: confidence interval; CR: complete response; CSC: cancer stem cell, Cy: cyclophosphamide; DC: dendritic cell; DCR: disease control rate; (m)DFS: (median) disease-free survival; (m)DOR: (median) duration of response; DTH: delayed-type hypersensitivity; 5-FU: 5-fluorouracil; GM-CSF: granulocyte monocyte-colony stimulating factor; GVAX: GM-CSF-based whole cell vaccine; HR: hazard ratio; HSV: herpes simplex virus; IFN: interferon; KIF20A: kinesin family member 20A; (K)RAS: (Kirsten) Rat Sarcoma Virus gene; (m)DFS: (median) disease-free survival; MHC: major histocompatibility complex; mo: month; (m)OS: (median) overall survival; (m)PFS: (median) progression-free survival; (m)RFS: (median) recurrence-free survival; MUC1: mucin 1; NK-cell: natural killer cell; NR: not reached; ORR: objective response rate; PBMC: peripheral blood mononuclear cells; PD: progressive disease; PDAC: pancreatic ductal adenocarcinoma; Poly-ICLC:polyinosinic-polycytidylic acid stabilized with polylysine and carboxymethylcellulose; PR: partial response; pts: patients; SD: stable disease; VEGFR; vascular endothelial growth factor receptor; VRP: virus-like replicon particles; y: year.

## Data Availability

Not applicable.
